# Supplementary material for: Development of a pentavalent broadly protective nucleoside-modified mRNA vaccine against influenza B viruses
Source: Nat Commun. 2022 Aug 9;13:4677. doi: 10.1038/s41467-022-32149-8 (PMC9362976; doi:10.1038/s41467-022-32149-8)
Supplement: Supplementary file 1 — Supplementary Figures [file 41467_2022_32149_MOESM1_ESM.pdf]

Supplementary information

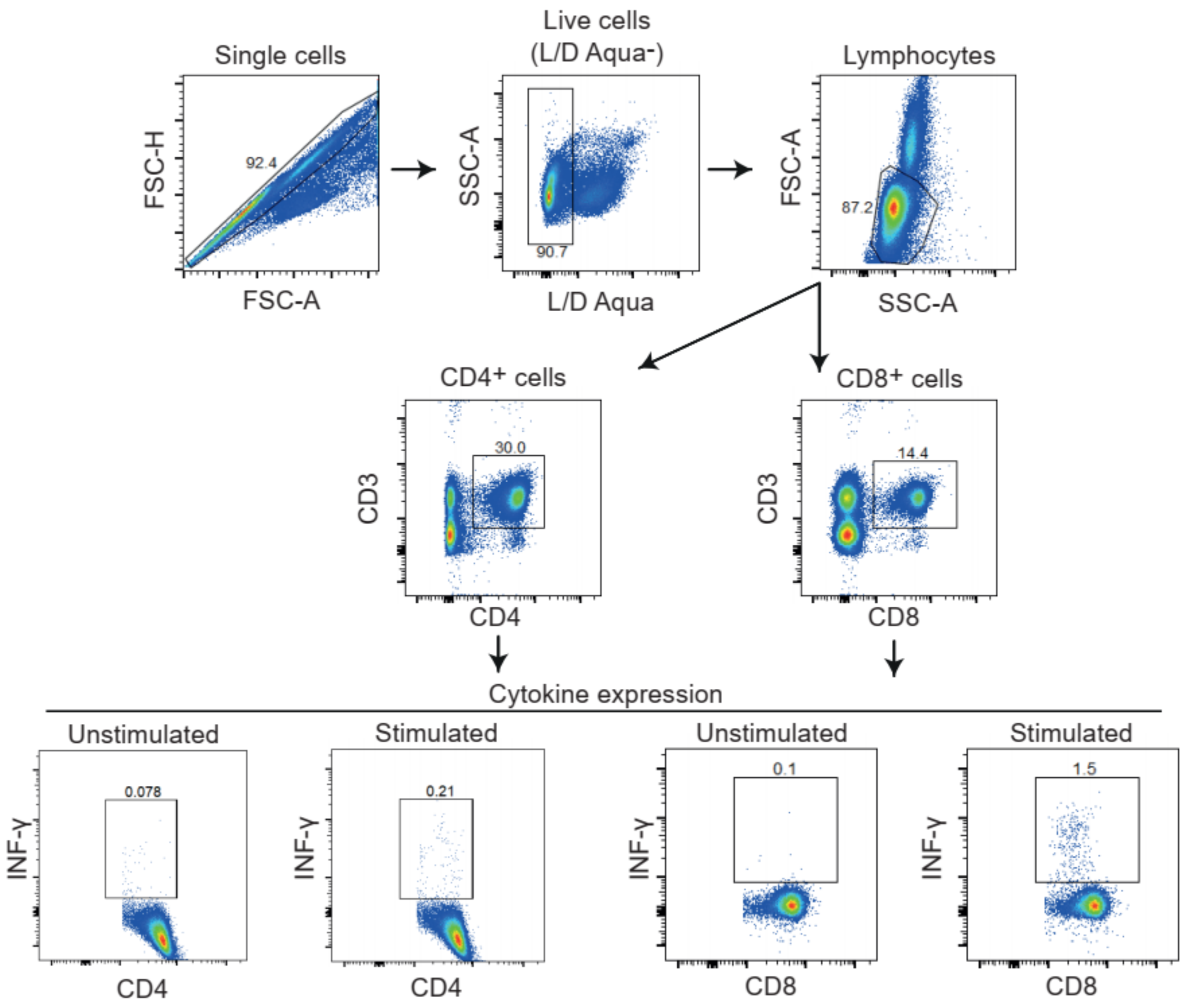

Supplementary Fig. 1 Flow cytometric gating strategy for the investigation of T cell responses in IBV mRNA-LNP-immunized mice. Representative flow cytometry plots for unstimulated and peptide-stimulated samples are shown. Source data are provided as a source data file.

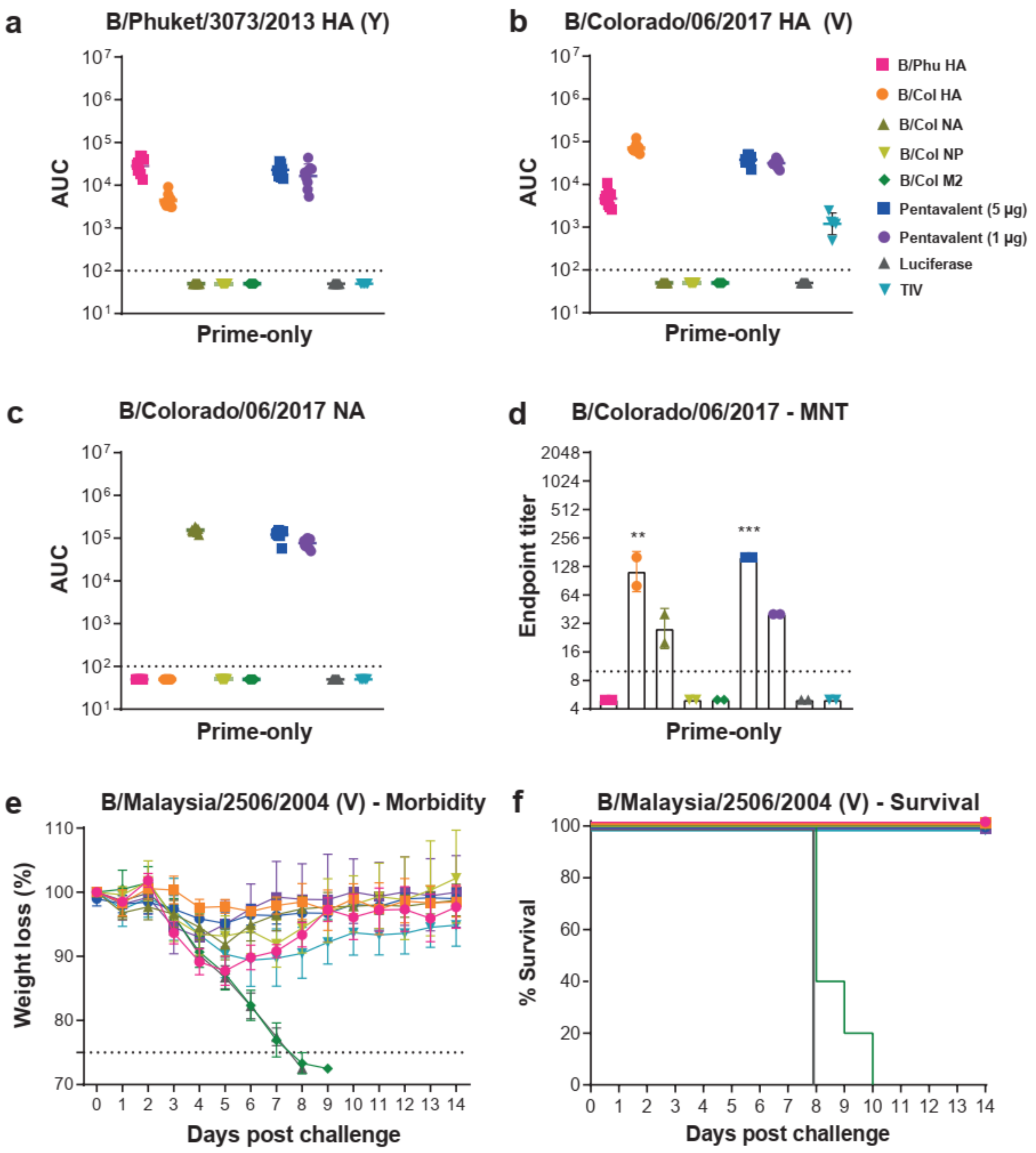

Supplementary Fig. 2 Comparison of the performance of influenza mRNA-LNP vaccines to a matched trivalent influenza virus vaccine. Mice were vaccinated I.D. once with monovalent mRNA-LNPs (5 µg) or with 5 µg/antigen or 1 µg/antigen of the pentavalent mRNA-LNP formulation. Control animals received 5 µg of Luc mRNA-LNP I.D. or a trivalent influenza virus vaccine I.M. (TIV, Fluzone, 2006-2007) at 1 µg of matched HA. Sera were collected on day 28 post single immunization and binding of antibodies to influenza virus antigens was measured by ELISA. Binding of sera against B/Phuket/307/2013 HA (n = 10) **a**, B/Colorado/06/2017 HA (n = 10) **b**, or B/Colorado/06/2017 NA (n = 10) **c** is shown. Each symbol represents one animal. Neutralization against B/Colorado/06/2017 using pooled sera from every group of mice in duplicate is shown (p values left to right 0.0013 and 0.0002) **d**. Morbidity and survival after I.N. challenge with 5mLD50 of B/Malaysia/2506/2004 (V) virus are shown (n = 4 or 5 mice/group) **e, f**. **a-d** The dotted lines indicate the limit of detection. **e** The dotted lines indicate the maximum weight loss (25%) for the experiment. For ELISA, AUCs

with a cutoff value of the average background plus three SDs are shown. For all data, mean with SD is shown. Significance was assessed using a one-way ANOVA and groups were compared to the Luc control group. * = $p < 0.05$, ** = $p < 0.01$, *** = $p < 0.001$, **** = $p < 0.0001$. Source data are provided as a source data file.

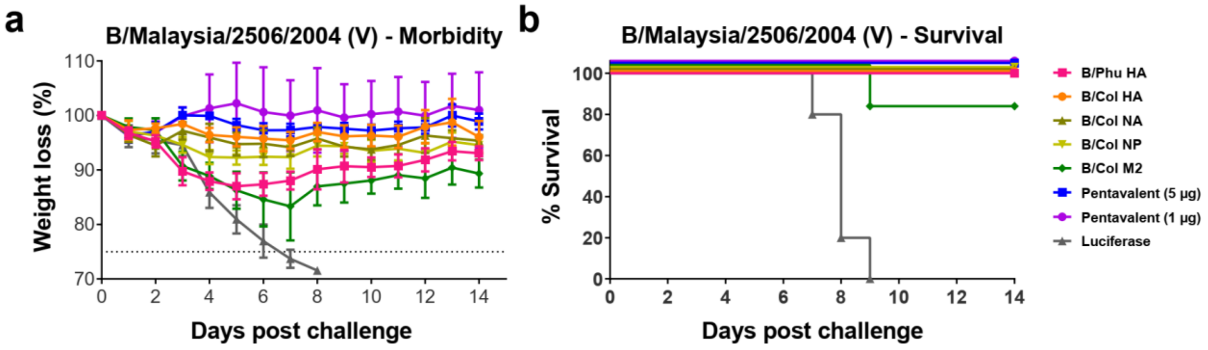

Supplementary Fig. 3 Morbidity and survival graphs in mice challenged with B/Malaysia/2506/2004 (V) influenza virus following prime-boost vaccination. B/Malaysia/2506/2004 (V) influenza virus challenge in mice vaccinated I.D. with 5 µg of monovalent mRNA-LNPs or 5 µg/antigen or 1 µg/antigen of pentavalent formulations in a prime-boost vaccination regimen where the prime and boost were separated by 28 days. Mice were I.N. challenged with 5mLD$_{50}$ of B/Malaysia/2506/2004 (V) influenza virus at 28 days post boost and morbidity **a**, and mortality **b** were measured for 14 days post challenge. Data are shown as mean with error bars representing SD (n = 5 mice per group). The dotted lines in a indicate the maximum weight loss (25%) for the experiment. One mouse was excluded from the luciferase group due to failed challenge (no weight loss observed). Source data are provided as a source data file.
