# Supplementary material for: Development of a pentavalent broadly protective nucleoside-modified mRNA vaccine against influenza B viruses
Source: Nat Commun. 2022 Aug 9;13:4677. doi: 10.1038/s41467-022-32149-8 (PMC9362976; doi:10.1038/s41467-022-32149-8)
Supplement: Supplementary file 3 — Reporting Summary [file 41467_2022_32149_MOESM3_ESM.pdf]

nature portfolio

Corresponding author(s): Meagan McMahon, Norbert Pardi, Florian Krammer

Last updated by author(s): Jul 3, 2022

Reporting Summary

Nature Portfolio wishes to improve the reproducibility of the work that we publish. This form provides structure for consistency and transparency in reporting. For further information on Nature Portfolio policies, see our Editorial Policies and the Editorial Policy Checklist.

Statistics

For all statistical analyses, confirm that the following items are present in the figure legend, table legend, main text, or Methods section.

n/a	Confirmed	
☐	☒	The exact sample size (n) for each experimental group/condition, given as a discrete number and unit of measurement
☐	☒	A statement on whether measurements were taken from distinct samples or whether the same sample was measured repeatedly
☐	☒	The statistical test(s) used AND whether they are one- or two-sided<br>*Only common tests should be described solely by name; describe more complex techniques in the Methods section.*
☐	☒	A description of all covariates tested
☐	☒	A description of any assumptions or corrections, such as tests of normality and adjustment for multiple comparisons
☐	☒	A full description of the statistical parameters including central tendency (e.g. means) or other basic estimates (e.g. regression coefficient) AND variation (e.g. standard deviation) or associated estimates of uncertainty (e.g. confidence intervals)
☐	☒	For null hypothesis testing, the test statistic (e.g. F, t, r) with confidence intervals, effect sizes, degrees of freedom and P value noted<br>*Give P values as exact values whenever suitable.*
☒	☐	For Bayesian analysis, information on the choice of priors and Markov chain Monte Carlo settings
☒	☐	For hierarchical and complex designs, identification of the appropriate level for tests and full reporting of outcomes
☒	☐	Estimates of effect sizes (e.g. Cohen's d, Pearson's r), indicating how they were calculated

Our web collection on statistics for biologists contains articles on many of the points above.

Software and code

Policy information about availability of computer code

Data collection	A BD LSRII flow cytometer with FACS Diva software (Version 8.0.1) was used for collecting flow cytometry data
Data analysis	Amino acid sequences were analyzed using the Clustal Omega multiple sequence alignment tool (https://www.ebi.ac.uk/Tools/msa/clustalo/). The sequence alignments were performed in September 2021. The percent (%) amino acid identity was determined using the computed percent identity matrix and examined for each challenge virus.<br><br>Flowjo10 was used for analyzing flow cytometry data.<br><br>All statistical analyses were performed using GraphPad Prism 8.

For manuscripts utilizing custom algorithms or software that are central to the research but not yet described in published literature, software must be made available to editors and reviewers. We strongly encourage code deposition in a community repository (e.g. GitHub). See the Nature Portfolio guidelines for submitting code & software for further information.

Data

Policy information about availability of data

All manuscripts must include a data availability statement. This statement should provide the following information, where applicable:

- Accession codes, unique identifiers, or web links for publicly available datasets
- A description of any restrictions on data availability
- For clinical datasets or third party data, please ensure that the statement adheres to our policy

Source data are provided with this paper.

Field-specific reporting

Please select the one below that is the best fit for your research. If you are not sure, read the appropriate sections before making your selection.

☒ Life sciences ☐ Behavioural & social sciences ☐ Ecological, evolutionary & environmental sciences

For a reference copy of the document with all sections, see nature.com/documents/nr-reporting-summary-flat.pdf

Life sciences study design

All studies must disclose on these points even when the disclosure is negative.

Sample size	Experiments were performed with at least 4-5 mice in each experimental group based on our extensive previous experience with mRNA-based influenza vaccines. Experiments assessing virus titres in the lungs of mice only contained 3 mice/group due to the complexity of harvesting organs from multiple groups of mice.<br><br>Assessment of antibody responses in serological assays involved at least 3-5 mice. Serum for these assays were randomly selected on the day of the assay. These numbers are based on our previous experiments with these serological assays following influenza vaccination in mice.
Data exclusions	No data were excluded from assays
Replication	Challenge experiments were performed once with 4 or 5 mice/challenge virus to reduce the number of mice used in these studies based on the 3Rs of animal research. For serological assessment experiments were performed once. T cell studies were performed with 7-8 animals in each experimental group.
Randomization	Animal cages were randomly assigned to vaccination groups. For serological assessment where only a subselection of sera were assessed, samples were randomly selected for analysis.
Blinding	Investigators were not blinded in our studies. The investigators who designed the experiments also performed the experiments so blinding was not possible.

Reporting for specific materials, systems and methods

We require information from authors about some types of materials, experimental systems and methods used in many studies. Here, indicate whether each material, system or method listed is relevant to your study. If you are not sure if a list item applies to your research, read the appropriate section before selecting a response.

Materials & experimental systems

n/a	Involved in the study	
☐	☒	Antibodies
☐	☒	Eukaryotic cell lines
☒	☐	Palaeontology and archaeology
☐	☒	Animals and other organisms
☒	☐	Human research participants
☒	☐	Clinical data
☒	☐	Dual use research of concern

Methods

n/a	Involved in the study	
☒	☐	ChIP-seq
☐	☒	Flow cytometry
☒	☐	MRI-based neuroimaging

Antibodies

Antibodies used	All antibodies (with cat# and vendors) are detailed in the methods section of the manuscript.
Validation	Dilution studies with CR8033, CR8059 and 1G05 were conducted with HEK293T transfected cells for optimal staining in flow cytometry.

Eukaryotic cell lines

Policy information about cell lines

Cell line source(s)	SF9 (CRL-1711) , MDCK (CCL-34) and HEK 293T (CRL-3216) cells were all obtained from ATCC. High-Five (B85502) cells cells were obtained from ThermoFisher. ADCC effector cells obtained from Promega.
Authentication	None of the cell lines were authenticated.
Mycoplasma contamination	None of the cell lines were assessed for mycoplasma contamination.

Commonly misidentified lines (See ICLAC register)	No commonly misidentified cell lines were used.

Animals and other organisms

Policy information about studies involving animals; ARRIVE guidelines recommended for reporting animal research

Laboratory animals	Female BALB/c mus musculus were purchased from Jackson Laboratories or Charles Rivers Laboratories between 5-7 weeks of age and used in experiments between 6 -8 weeks of age.
Wild animals	The study did not involve wild animals.
Field-collected samples	The study did not involve field collected samples.
Ethics oversight	Mouse studies were conducted under protocols approved by the Institutional Animal Care and Use Committees (IACUC) of the University of Pennsylvania (UPenn) and the Icahn School of Medicine at Mount Sinai (ISMMS). All animals were housed and cared for according to local, state, and federal policies in an Association for Assessment and Accreditation of Laboratory Animal Care International (AAALAC)-accredited facility.

Note that full information on the approval of the study protocol must also be provided in the manuscript.

Flow Cytometry

Plots

Confirm that:

- [x] The axis labels state the marker and fluorochrome used (e.g. CD4-FITC).
- [x] The axis scales are clearly visible. Include numbers along axes only for bottom left plot of group (a 'group' is an analysis of identical markers).
- [x] All plots are contour plots with outliers or pseudocolor plots.
- [x] A numerical value for number of cells or percentage (with statistics) is provided.

Methodology

Sample preparation	Luc, B/Phu HA, B/Col HA, or B/Col NA mRNA-transfected HEK 293T cells were collected 24 hours after transfection and were washed once with 1% fetal bovine serum (FBS) (HyClone) in phosphate buffered saline (PBS) (Corning, DPBS 1X (without calcium and magnesium)). Next, cells were incubated with 7.5 μg/ml anti-NA (1G05) or 7.5 μg/ml anti-HA (CR8033 for B/Phu HA and CR8059 for B/Col HA) antibodies in V-bottom 96-well plates (Greiner) at 4°C for 30 min. After washing with 1% FBS in PBS cells were incubated with anti-human AlexaFluor-647 (goat polyclonal, 1:300, ThermoFisher, #A21445) secondary antibody at 4°C for 30 minutes in dark. The samples were washed and re-suspended with 1% FBS in PBS.<br><br>Spleen single-cell suspensions were made in complete RPMI 1640 medium (ATCC). 3X106 cells per sample were stimulated for 6 h at 37°C and 5% CO2, in the presence of overlapping NP (BEI Resources, NR-19254), NA (BEI Resources, NR-19254), HA (BEI Resources, NR-18972) peptide pools at 2.5 μg/mL per peptide. GolgiPlug (5 mg/mL; brefeldin A; BD Biosciences) and GolgiStop (10 mg/mL; monensin; BD Biosciences) were added to each sample after 1 hour of stimulation. Unstimulated samples for each animal were included. A phorbol 12-myristate-13-acetate (10 mg/mL; Sigma) and ionomycin (200 ng/mL; Sigma)-stimulated sample was included as a positive control. After stimulation, cells were washed with PBS and stained for 10 min in dark at 25°C with the LIVE/DEAD fixable aqua dead cell stain kit (Life Technologies). Samples were incubated in the Fc blocker (Purified Rat Anti-Mouse CD16/CD32) for 10 min in dark at 4°C and then surface-stained with the monoclonal antibodies anti-CD4 PerCP/Cy5.5 (BioLegend) and anti-CD8 Pacific Blue (BioLegend) for 30 min at 4°C. After surface staining, cells were washed with FACS buffer, fixed and permeabilized using the Cytofix/Cytoperm kit (BD Biosciences). Cells were intracellularly stained with anti-CD3 APC-Cy7 (BD Biosciences), anti-TNF-α PE-Cy7 (BD Biosciences) anti-IFN-γ AF700 (BD Biosciences), and anti-IL-2 BV711 (BioLegend) mAbs for 30 min at 4°C. Next, the cells were washed twice with the permeabilization buffer (BD Biosciences), fixed with 4% paraformaldehyde in PBS, and stored at 4°C until analysis. Splenocytes were analyzed on a LSR II flow cytometer (BD Biosciences). 200,000-500,000 events were collected per specimen. Data were analyzed with the FlowJo 10 program. Data were expressed by subtracting the percentages of the unstimulated stained cells from the percentages of the peptide pool-stimulated stained samples.
Instrument	BD LSR II flow cytometer.
Software	BD FACS diva software for collection, FlowJo 10 for analysis.
Cell population abundance	At least 50,000 cells after cell transfections and 200,000-500,000 cells in T cell studies were collected.
Gating strategy	Total populations are presented.

- [x] Tick this box to confirm that a figure exemplifying the gating strategy is provided in the Supplementary Information.
